# Dr. Beale's Case

**Published:** 1855-01

**Authors:** 


### ARTICLE IX.

### *Dr. Beale's Case.*

THE excitement produced by the very remarkable trial of Dr. Beale, of Philadelphia, has been such, that some notice of it will naturally be expected from our Journal. We had intended to prepare a review of the testimony, based upon the report published in the New York Police Gazette, which is generally admitted to be a fair and full statement of the entire proceedings had in the case; but we find, in the Medical Examiner, of Philadelphia, an article by Dr. Hartshorne, and another by Dr. Stillé, which, taken together, form so complete a review of the whole subject, that we have thought we could not do better than present them, unmutilated, to our readers. We do not see how any unprejudiced mind, after a careful perusal of the whole testimony and the facts and arguments contained in the two following papers, can come to any other conclusion than that arrived at by their able authors.

We cannot, however, consent to let even these appear without some direct and decided expression of our own views. We must premise, that, in the remarks we are about to make, we are influenced by no prejudice or partiality. The writer of these lines is altogether unacquainted with the parties in the case, and he believes he can say the same for both his colleagues. As for himself, he was alike ignorant of the existence of Dr. Beale and Narcissa Mudge, before this affair dragged them both into such unenviable notoriety. We know nothing and care nothing for Miss Mudge's position in society, and in what little we have to say, we shall treat her as we would any other witness in a criminal case, attaching just so much importance to her statements as they show themselves entitled to, and no more.

The first thing we have to say, is to call attention to the well known rule of evidence; the greater the antecedent improbability of any declared fact, the more direct, positive and indubitable must be the testimony by which such a fact is proposed to be established. Thus, if a man entrusted with money were to say that he was knocked down and robbed in broad day-light, in the crowded thoroughfare of a great city, he could only save his credit for veracity and honesty by bringing the most irrefragable proof of the truth of his assertion. A jury of madmen or idiots, might believe his individual oath, but we doubt if twelve men, possessed of the average allowance of common sense, could be found to make up their minds upon such testimony.

Now let us look at the antecedent improbability in the case before us. A man of family, living on good terms with his wife, of standing in society, and dependent upon that standing for his livelihood, is charged with having committed a rape upon a young girl. There is nothing peculiar about the circumstances to rouse the lustful passions of this man; on the contrary, he has known her from her childhood. She is a familiar acquaintance. She has not dazzled him with any sudden beauty, but seems to have been a common-place girl, surrounded by very prosaic circumstances. Whence then this rapacious appetite for such ordinary charms? Still, all this is possible. There is no accounting for the whims of men when women are concerned. There is no sounding the abysses of beastliness in some hearts.

But granting the possibility of this lecherous appetite, capable of overcoming every better emotion of a decent man, decent at any rate so far as reputation is concerned, and having the strongest possible motives for maintaining that character; granting all this, how would such a man have proceeded to the gratification of his vicious inclinations? Absaloms, who commit adultery on the house-tops, are not to be found among prudent, thriving men of the world. They know the value of the outer whitewash too well for that, and take care to do their dark deeds in darkness. A man, in the circumstances of this unfortunate dentist, would have proceeded very differently. He would have taken every precaution against detection. He would have selected an hour, when no one was about, when there was no likelihood of interruption, and he would have taken especial care that his victim should, in one way or other, have her mouth closed against him. Such, at any rate, are the probabilities in the case. A man might act differently, but to show that he did would require a formidable array of proof.

Now how is this man, to whom his character represents so many dollars and cents in hard cash, accused of having acted? Why, of having made a public appointment to meet her in a public place, in broad day-light, in his own house, in a room overlooked from all quarters, open to his own domestics, habitually resorted to by his workmen, at an hour when he was likely to be disturbed by other visitors, under circumstances in every way unfavorable to the purpose he designed to accomplish. The idea of a deliberate purpose is so manifestly absurd, that the prosecution did not attempt to press it.

There remains, therefore, but one hypothesis, which is that the appointment was made by both parties, in good faith, and that the rape was a sudden thought, suggested by seeing the young girl in his power, under the influence of an anæsthetic. This is the only possible ground to take, and this accordingly, is the position assumed by the prosecution. Here again, however, we are met by hosts of improbabilities. This man is not an amorous youth, full of passion and deprived of the means of gratification; he is a man of middle age, for all that appears, happily married. He is no libertine, accustomed to an unbridled indulgence of his passions, so that from long habit he has become their absolute slave; but a sedate man of business, a member of a proverbially strict religious society and largely dependent, for his daily subsistence, upon the good character he had labored to acquire. Now, we know very well that it is customary to sneer at religious people, and that a vast deal of Bowery cant is always lavished on an occasion like the present. But we care nothing at all about the sincerity of Dr. Beale's religious profession. He may be saint or sinner, zealot or hypocrite, it is not material to our argument to classify him. We wish to look upon the matter solely in a worldly point of view, and it really seems to us that it strengthens his case to suppose him a hypocrite. For, if he was the man of such strong natural passions as this woman's statement implies, he must have put a strong and habitual control upon them, to have maintained his position in the church, and must have had most powerful inducements to have assumed such a position in the outset. Don Juan or Lord Rochester would not become a puritan but under the influence of uncommonly strong motives. The most probable reason for such a step, appears to us the desire of extending his business, an influence, which of course, must continue as long as the power of attending to that business lasted, and must have been quite as powerful at the time of the alleged crime as at first. Under any circumstances, he must necessarily have established such a control over himself as to render it exceedingly improbably that he would commit so rash an act, so open to detection, and thus, in one moment of passion, shatter the fabric he had been erecting with such continued labor and habitual self-denial.

And now for the physical improbability. Look at a dentist's chair; we write for dentists; and tell us what sort of facilities does it afford for a rape? Dr. Parmly saw the identical chair and stool, and has published it as his deliberate opinion, that such an act there was (not improbable, but) impossible.

What then is the testimony by which Miss Mudge proposes to establish the fact of her defloration? We do not wish to go over all the disgusting details into which this modest young lady enters with such remarkable minuteness. The astonishing self-possession which could, at so critical a moment, take cognizance of such minute particulars can only be paralleled by the amazing calmness with which this astounding wrong was received. Messalina herself could not have exhibited more decided indifference to a violation of her personal sanctity. She does not, as any virgin we know would have done, burst out into an agony of tears, and wander distractedly away from the accursed scene of her pollution, or dilating into a Nemesis, strike down the foul author of so atrocious a wrong. Nothing of the kind, she converses amicably with him, appoints another meeting, allows him to introduce her to another patient, takes leave of him pleasantly, goes to an ice cream saloon, chats with a friend, orders a dress, takes a ride for pleasure in the afternoon, &c. This certainly is a new phase in the mental character of a recently debauched virgin. Is it not highly improbable that any woman could be so calm after such a scene?

But to her testimony! She tells us that after she was etherized, Dr. Beale proceeded to take certain liberties. She describes, with unblushing minuteness, all the preliminaries to the final act, and so far every thing is well enough, save that the presence of sensation and the absence of volition do not harmonize very well. But here we reach most remarkable discrepancies, which we are surprised that any jury could overlook. She has sensation, and she don't have sensation—she feels in her cheek, she feels in her arm, in her bosom, in her legs, but she dont feel in that part of her body about which is the whole controversy. How is it possible for such irregular anæsthesia to exist? What a remarkable distribution of nerves must Miss Mudge be endowed with! What a godsend would such a nervous system be to a miracle-hunting physiologist. For observe, she expressly says that she feels Dr. Beale's breath upon her cheek, his hands upon her bosom, arms and legs, but not his body against her body. She knows that he pulls up her clothes, that he arranges her limbs in the most skillful manner; she is perfectly conscious of all these preliminaries, but she does not feel the man in the very act of which she complains. How is any jury to reconcile these discrepancies?

Another general principle of law must also be remembered. It is not for Dr. Beale to prove his innocence, but for the prosecution to establish his guilt. If they fail in that, the whole case falls to the ground, and the man guilty or not, must be liberated. All that is necessary, is to show us not that he is *innocent*, but that he has not been *proved* guilty.

Now what is rape? The carnal knowledge of a woman against her will. But what is the carnal knowledge of a woman? Here the authorities differ—some tell us that penetration and emission are both necessary, others that the smallest amount of penetration is sufficient. In this case, however, the distinction is not of the slightest consequence, because no interruption having taken place, we are bound to suppose the act perfect, and penetration and emission both complete.

Where then, we ask, is the evidence? By the most surprising neglect, if indeed that be the proper term to apply to it, no examination whatever was had of the linen of the young woman, so that there was no evidence whatever of emission, and the prosecution attempted only to prove penetration.

But how is this made out? Miss Mudge is satisfied that it took place—how does she know it? By her own confession, she did not feel Dr. Beale's person against her person. All she swears to positively is the sense of pain somewhere in the organs of generation. She admits that she was near the period of menstruation, and we all know that in most women that period is attended with some pain in this region. What then more likely than that under the influence of an amorous dream, induced by the ether, she should have referred this sensation to the act which was present to her imagination?

One of the most painful features in this whole affair, is the absolute indifference to medical evidence exhibited by the judge and jury in this case. Did this woman leave Dr. Beale's a virgin? Who knows? No one has taken the trouble to inquire. Were there traces of semen upon her clothes, or were they totally devoid of such evidences of pollution? Here again we are entirely at sea.

Still further, supposing the carnal knowledge established, where is the evidence that it was done against the woman's will? According to her own confession, she made no outcry, and *did not try to make one*—does not know whether she had the power to make one; but she did scream, after the second dose of ether, when her tooth was pulled. This is a very remarkable statement, and ought to have been carefully weighed by the jury. It cannot be said that the influence of the ether was passing off when the tooth was pulled, because she acknowledges to have taken a second dose. The time at which she was least under the influence of the anæsthetic, was during the very act of which she complains, for it was immediately after that, that the second dose was administered. If it be said, that the pain of drawing the tooth was greater than that of the rupture of the hymen, in consequence of the relaxation of the parts; the reply is first, that such relaxation is not proved to attend etherization; and, secondly, that the moral shock of a rape would be quite as likely to rouse a virtuous woman, as any physical pain whatever.

We have thus seen that there is not a shadow of external circumstantial evidence to corroborate Miss Mudge's statement, and we find it impossible to believe that statement; first, because her own conduct after the affair is totally inconsistent with the story she tells, it being, in our opinion, impossible for a woman to bear such an indignity so calmly. It is idle to say, that she feared Dr. Beale, that she did not know what to do. That might have suppressed any manifestation of anger, but it could certainly not have quieted the tumult of emotion in a virgin's bosom, at so gross and terrible an outrage.

Secondly, we disbelieve her statement, because it is inconsistent with itself, as we have endeavored briefly to show, and as will more abundantly appear on the perusal of the following articles.

Thirdly, we disbelieve her, because she was not in a condition to know positively the facts to which she swears. Suppose Miss Mudge were to have told the jury that, while at Dr. Beale's, she was so drunk as to be impossible, to offer any resistance to him. Who would have believed her? It would not then have availed, to offer to prove that some drunken people recollect what happens to them during the temporary alienation of their reason, because it is notorious that the chances are against any such self-possession and clearness of memory. The majority have drunken dreams, and fancy that things have happened to them, which, in reality, did not take place. If such a witness makes a statement, men of sense compare his account with facts arrived at independently of him, and form their judgment accordingly. Now what is etherization but a species of intoxication, and how can we make a distinction between it and any other kind, that will hold good in the discussion of a point like this? As well might we be willing to believe the statements of a man drunk on wine, and refuse to believe those of another, intoxicated with whiskey. There are no collateral circumstances to corroborate Miss Mudge's statement, and hence, in the nature of the case, we cannot see that there is any evidence at all.

In reply to all these doubts, we are pointed to this lady's position in society, and asked, what possible motive she could have had for falsifying facts. In reply, we have only to say, that we cannot see the propriety of requiring one who does not believe a story on the ground of its inherent improbability, and irreconcilable discrepancies, to suspend his opinion till he can discover the motive for the falsehood. There are many motives that might induce a woman so to act, which will occur to any one upon a moment's thought. In this case, however, it is unnecessary to seek for motives. Every one knows that the feeling of the moment is that which is likely to manifest itself during ethereal intoxication, and there seems to be reason for believing that ether has some influence in awakening the sexual appetite. We have, therefore, only to suppose an amorous dream under the influence of ether, shame on subsequent reflection upon it, the confused recollections of intoxication and the peculiar resentment consequent upon such recollections, and the whole thing is accounted for.

From this point of view, Miss Mudge cannot be blamed, but the jury who listened to her story, without seeing its discrepancies, or caring for the absence of medical or other corroborating testimony, are certainly highly censurable.

We have studiously forborne making any comments upon the behavior of any of the parties in this case. We have confined ourselves solely to the recorded proceedings, and feeling no partiality or prejudice towards any one, have avoided all purely personal allusions. This was not because the case did not afford ample scope for grave objections to personal acts, but because we preferred to discuss exclusively the facts and principles involved.

### *Remarks on the Case of a Dentist, Convicted of Violating a Patient while under the Influence of Ether Inhalation.* By E. Hartshorne, M. D.

The case of the dentist recently on trial in this city for an alleged outrage on the person of a patient while under the influence of inhaled ether has painfully attracted the attention of our whole community, notwithstanding the revolting nature of its details. Fortunately for the peace of that community, and the reputation of anæsthetic agents, it is without a precedent in this country or Great Britain, and has but a solitary counterpart elsewhere. It has inflicted a lasting shock upon the public sentiment in relation to the use of anæsthesia in the absence of third parties and for trivial operations; and were it not for the dreadful stigma cast upon accuser and accused, and the lifelong blight entailed upon the latter, as well as all connected with him, we might try, as professional observers, to look upon the whole affair as a salutary, though bitter, warning. But the inconclusive and meagre investigations and unhappy termination of the trial, have rendered it a source of serious distrust to all reflecting medical inquirers. It was not surprising that the hue and cry of popular excitement, and their natural echo in the columns of the public press, should strive to cut the Gordian knot without regard to scientific truth, but we confess to a grievous disappointment in the result of the legal discussion of the charge.

We have no desire to impeach the peculiar verdict of the jury, but we are imperatively called upon, as medical journalists, to engage at once, and to the best of our abilities, in the medico-legal consideration to the question. The vital importance of a full and free inquiry of this kind to the best interests of our profession as well as of society in general, must serve as our excuse for undertaking such a thankless and laborious duty. The task is anything but an inviting one, and we would cheerfully resign it into other hands more qualified to do it justice. Still less do we incline to add fuel to the flame of morbid curiosity already stirred up and fuming, *usque ad nauseum*, under the stimulus of the recitals of the crowded court room, and the ample though prudely mutilated pictures of the daily papers. This very publicity, however much to be deplored and deprecated, increases the necessity for a closer, and we hope more thorough scrutinizing of the subject.

In thus dealing with the facts and reasonings presented by the only available reports of the trial, our intention is to adhere as much as practicable to the medical facts and principles alone. We have no personal acquaintance with the plaintiff or defendant, and do not profess to be the advocate of either party. Our desire is for the sake of our medical brethren, to whom this paper is especially addressed, honestly to arrive at what we conceive to be the truth so far as that can be really ascertained; at all events to release the record as it now stands before us, from some share of what experienced medical jurists will support us in asserting to be dangerous error.

In entering upon our statement of the case, we take occasion to mention that the report from which we quote is that published in the National Police Gazette. It is the fullest we have seen, and is sufficiently complete and accurate we hope to serve the present purpose.

A young lady of unimpeachable character who has for some time been engaged to be married, is accompanied by her betrothed to the house of an eminent and highly respectable dentist, who had engaged to plug one of her teeth. They arrive about 10 o'clock on a Friday morning. She enters the house, and after "a few minutes" spent in awaiting the exit of two other ladies, she is ushered into the operating room or office. Here we will allow her to continue the narration in her own words.

I went into the office; took off my bonnet, and Dr. B—— went to the washstand to wash his hands, and he asked me after the family; I took a seat on the operating chair; in a few minutes Dr. B—— told me one of the men wanted to speak to him, and he gave me a book to read and left the room; did not say what man; I supposed there were men there; he has a room in which the teeth are made; I believed those to be the men; Dr. B——'s family were out of town at that time; he said so, and the door was opened, and there was no furniture in the front room; I don't know how long Dr. B—— was absent; when he came back I was sitting in the operating chair; he went to the instrument case, and began with my tooth; the tooth was on the left side; he commenced operating on the tooth before he gave me ether; the operation was very painful; he said he would either put something in to destroy the nerve, or give me ether, leaving the choice to me; I told him I'd prefer taking ether; I didn't learn what he proposed putting into the tooth; he gave me the ether on a small napkin, folded up; I felt very dizzy at first; I was cold and felt very numb; it increased upon me; I did not lose my consciousness of what was doing; I continued to breathe the ether; my eyes were closed; I closed them voluntarily; I did not try to open them for some time after; after he gave me the ether he did not, as I remember, operate on my tooth; he felt my pulse several times; put his hand on my arm under my sleeve, up my arm; I had a loose sleeve; he did it once; he put his hand on my breast under my dress, on the bosom; he put his hand on my person, under my dress; I have a distinct memory of that; I was not able to make any resistance or outcry; he went round before me and raised my clothes; I am perfectly distinct in my memory of that; I did not try to cry out; do not know if I was able; after he had raised my clothes, my feet were crossed, and he raised them and put one on each side of the stool; he then put his arm around me under my clothes; he drew me down to the edge of the chair; I do not know what he did after that till I felt pain; he did enter my person; it was then I felt the pain; I was not able to cry out or resist; I did not try; I don't know what was his position; my eyes were closed; I have no doubt that he did enter my person, and did give me pain; all this time I was conscious of every thing that was going on; after this he left me and crossed the room to the washstand; I heard him pour out water into the basin; after he had been to the washstand and returned, I opened my eyes, and saw my clothes up; he did not see me; I have a clear recollection of seeing my clothes up; I closed my eyes immediately; he put down my clothes, and in a few minutes he was at the side of the chair, and lifted me up into the seat; I was just to the edge of the seat; it was a large dentist chair; in a few minutes he told me he'd have to take the tooth out; that was the first remark he made, except the first, when he asked me if I was getting sleepy; at the time he entered my person I did not feel his person against me; pain I distinctly felt; when he spoke about taking out the tooth, I asked him why? he said they were both decayed, and he could not save them both; I told him I was afraid it would pain me, and he said he would not let it; he then gave me more ether, and extracted the tooth; I was on the left side; when he extracted the tooth it was painful; I screamed then; he then assisted me to rise, and led me to the rocking chair; I felt a little dizzy when he led me to the rocking chair; he then went out of the room, and in a few minutes came up with a lady; I have not seen her since; he asked me if I would be introduced to her; I believe I said no; he did not introduce me then; I heard him tell the lady he'd always been our dentist, and that we never had been to any other; he said my teeth were very good; he said I had taken ether, when the tooth was extracted; I think she said something about hearing me scream; he said yes, ether had not much effect on me, I was either nervous or for some cause; in a little while I got up, and he introduced me to the lady; I think it was Mrs. P——; I made several remarks, but I don't know what they were; I then put on my bonnet, and Dr. B—— followed me down stairs; the lady was left up stairs; he came to the door, and I wanted to stop an omnibus; he asked me how far I was going, and I told him to Third street and Lombard; he told me I had better walk; he said he thought I had some of the ether in me, and the walking would do me good;

I walked down Walnut to Sixth, and did not get in an omnibus; I did not reproach Dr. B—— at the house; I was afraid; I stopped in Cane's ice cream saloon, at Sixth below Prune; I got ice cream; I went then along Sixth street to Spruce, and down to Third and Lombard street; I was going to see a young woman that sent for me; I did see her; don't recollect how long I was there; when I left I came up to Mr. T——'s, at Chesnut street, near Fifth; I was very intimate with Mr. and Mrs. T——; I met Mr. M—— on the way up, near Sixth and Chesnut street; he joined me and spoke to me; did not accompany me to Mr. T——'s; did not meet any but those I have named; I reached Mrs. T——'s at 1 o'clock; they had not been to dinner; I first mentioned to Mrs. T—— what had occurred at Dr. B——'s; the same day after tea; that afternoon I was taken unwell; it was the usual time; the door of the dentistry room at Dr. B——'s was shut; there are two doors in the room; the one leading to the entry door was closed; Dr. B—— said that he closed the door because the smell of the ether would go over the house; the door was shut before he gave me the ether; the chair is one that leans backwards.

Cross-examined—Dr. B—— was the dentist of our family; don't remember the number of years; it was from the time of my early youth; he attended to all the members of the family so far as they required it; I went to him with the approval of my parents; he generally behaved like a gentleman; I did not know his family; don't know how many years I have been his patient; when I called with Miss Thr——— it was to get my tooth plugged; on several times before I had taken ether; I requested it to be given; I don't remember of his persuading me from it; the tooth was not plugged when I was there with Miss Thr———; the following Thursday was appointed for future operation; I did not go on Thursday; Mr. Thr——— had the appointment made; I believe it was on Wednesday morning; I received a letter from him to that effect; I requested him to go in with me; he was there when the woman came to the door; I was shown into the front parlor; it was the usual place; it was but a few minutes before the ladies came down; Mr. B—— came down before; he said he had several young ladies up stairs, and would be down in a few minutes; I went into the usual operating room up stairs; the door opening into the front room was opened at the time; it was the back room of the main building I was in; the workshop is in the second story brick building; don't know how far from the room in which I was; it is not upon the same level; it is lower;

I don't know if I could see into the windows of the workshop from the window of the room in which I sat; when Mr. B—— went to see the workmen he gave me one of the monthly magazines; while I was in the room nobody came to the door, that I saw or heard; don't know of the doctor leaving that room; did not see any woman there except Mrs. P—— and the Miss H——; the windows were closed in the room, *i. e.* the sashes were down; no change was made in their condition while I was there, don't remember any one calling as a sitter, while I was there, and Dr. B——'s speaking of it; I did not know of Mrs. P——'s being in that house before she was brought up stairs; I don't remember speaking to Dr. B—— of the fan, and requesting him to give me ether; from the time I closed my eyes after the ether had been taken, I did not open them until after the liberties had been taken; I did not open my eyes until he returned from the washstand; what I have described is from what I have heard and did not see; I did not see any part of his person exposed, nor the application of any part of his person to me; don't know, except from the pain, what part of his person was applied to me; he passed his hand up my arm immediately after he had felt my pulse; after the ether was administered a second time no liberties were taken; I judge that he did not see me when I opened my eyes, because he was not in front of me; when he told me he would have to pull the tooth, I asked him why; the reason why I agreed to take the ether a second time was because I was afraid; I was not afraid to have my tooth taken out, or to be operated upon further; I don't know if either of my teeth were prepared for plugging; I suppose he touched the tooth he took out; that gave me pain; I told him I'd had the toothache; another appointment was made for Monday; at 2 o'clock; I asked him when I was to come again to have them finished, and he said at that time; I asked him that when I was going and had my things on; he booked it at my instance; I don't know if it was before Mrs. P—— came in or not; Dr. B—— did not say there was a sitter waiting for the chair; I did not see any one call to inform him of a sitter; I never notice such small things as that; don't know how long after he had finished the tooth that he went down for Mrs. P——; I did not remain more than five minutes; Mrs. P—— said she came from the country, and came to have her teeth attended to; Dr. B—— followed me down stairs; that is his custom, not only with me but with other ladies; when at the door I did not manifest any displeasure with him; I told the doctor

I wanted an omnibus; I believe I bid him good bye; soon after I got out of the door of the second story, I told him to say good bye to Mrs. P—— for me, as I had forgotten it; the chair I sat in was the one I had always used; there was but one operating chair in the room; Dr. B—— asked me if I ever rode on horseback; I said yes, sometimes; he said ride over and see us; I replied, perhaps I will; that was up stairs; on the way down to C——'s I did not meet any one I knew; I did not meet any one on my way to Third and Lombard streets; I told Dr. B—— I was going on an errand to Third and Lombard streets; it was an errand for my sister in respect to some articles of dress; I did not speak to her of the treatment I received; did not sit down very long; when I left Dr. B——'s I think it was a few minutes before or after 12 o'clock; I don't remember which; I don't know how long I was at C——'s; not long; reached Mrs. T——'s a little after 1 o'clock; Mr. M'K——, whom I met, asked after the family; I did not tell him where I had been; he only walked with me a short distance; I did not complain of any pain to Dr. B——, except the pain of my teeth; I don't remember how long the first application of the ether lasted; after I took it I felt no pain in my teeth; cannot describe the effect of the ether, except that it made me dizzy; I did not see the doctor at all during the operation of the first ether; I felt his breath as well as felt pain; the pain did not continue long; I had no other indication of the approach of my monthly discharge but that day; it occurred in the evening; I did not examine my person in the interval; nobody examined it between those times; I did not examine my garments; my mother did on Sunday afternoon; nobody before; those garments don't remain now as they did then; they are washed; I don't know when; I made the communication to Mrs. T—— after tea on Friday evening; I told Mrs. T—— before I became unwell; I gave evidence before the Mayor; don't know if the garment was washed before that; it was not washed till I went out home; during the time I was at Mrs. T——'s till I was taken unwell, no physician was sent for; I was never examined by a physician; on the afternoon of Friday I was out riding with Mr. and Mrs. T——; we set out about six; I do not know where we went; somewhere on the plank road; it was some time after I returned that I felt unwell; spoke to Mrs. T—— on that subject after tea; we had tea as soon as we came home from riding; Mrs. T—— told Mr. T——, and Mr. Thr—— asked me a single question about it; I answered it; and that was all I said; it was before I felt unwell that I told Mr. Thr—— about it; he remained as long as I did, and went to my grandmother's with me; on the next day I went out to the depot, but did not go to my father's; Mr. Thr——— accompanied me to the depot; I met Mr. and Mrs. T—— out there; I did not see my father or mother; I saw my father on Monday morning in Fifth street; at the time he left to go down stairs, I did not see if he opened the door or not; I was sitting with my back to the door; I don't know why I refused to be introduced to the lady when he first asked me the question; my father and Mr. Thr——— accompanied me to the Mayor; Mr. and Mrs. T—— and my two uncles were there; my father was there before I was.

Re-examined—I said that Dr. B—— generally used me like a gentleman; he said a year ago that he should like me for his second wife; he had a good many children, but they should not trouble me, as he would get nurses for them; I spoke of it at home to my mother and sisters; after the doctor took me out of the chair after the operation, all that I said was in answer to questions by him, or to remarks; the reason why I did make another appointment with him (Dr. B——) was that I did not want him to know that I knew anything of his conduct; I had not concluded what course to pursue.

Miss M—— was asked in reference to the question put to her by Mr. Thr———, which was objected to by Mr. Brown. The Court overruled the question.

By Mr. Brown—The remark of Dr. B—— about having me for a second wife was, I thought, spoken sportively; I thought it improper, but mother said not.

Sarah T—— sworn —I reside in Chesnut street, below Fifth; I am acquainted with Miss M—— and family; have known her from a child; she came at about a quarter to 1, and remained there till about 8 o'clock in the evening.

To the question, did Miss M——, on the evening of the 4th of August, state anything of an outrage perpetrated by Dr. B——, Mr. Brown objected.

The Court ruled it out, on the ground that it was leading.

Mr. Reed varied, so as to bring it within the ruling of the Court.

Mr. Brown objected. He held that it would not be admissible at any time, much less at the present; such a thing was never heard of. Hearsay testimony could not be admitted.

Mr. Reed said, that if his friend could have brought a single book to show that the doctrine he advanced was the law, it would be worthy of some consideration; but he could not do it. The fact of a cotemporaneous declaration made by the victim of its secret crime, had always been held admissible. Mr. Reed quoted from 9th Humphreys a case in point.

Mr. Brown, in reply, said that in all the elementary books the principle is settled beyond controversy or cavil. The statement of the prosecutrix could not be given in evidence. Mr. Brown quoted from 1st Phillips, in which it is ruled that the complaint of the prosecutrix may be given but not her detailed statement.

The Court overruled the question on the ground that the prosecutrix could not make testimony for herself. It was not admissible as substantial testimony, nor to corroborate at this point of the case. The District Attorney could simply ask if Miss M—— did make a complaint on the evening in question.

Mrs. T——, in answer to the question, said that Miss M—— did make complaint at the supper table.

Mr. T——, sworn—I have known Miss M—— from childhood; on the afternoon of the day in question Miss M—— made a complaint to me of Dr. B——; Mr. Thr—— and my wife were present; it was at the supper table.

Mr. Thr—— also testified to the same points; after which, the Court adjourned.

The foregoing extract contains all the material testimony against the prisoner. On the part of the defence, in the first place, evidence of good character was abundantly presented. With this, we have nothing to do, as it does not affect the medical relations of the case. In the second place, the peculiarly exposed position of the operating chair, and the liability to frequent sudden interruption, were fully shown. This, also, forms a part of the moral evidence which does not come within the scope of a strictly medico-legal investigation. A question, also, of time is determined by this point of testimony, which is important, and shall be noticed in its proper place. Now comes the testimony of the lady who arrived at the close of the momentous interview. It describes the apparent calmness of both the operator and his patient.

"On leaving the room she asked the Doctor when she would call again; he named Monday, but I don't remember the hour; she said then, I will not go out of the city, as I am engaged to go out with a party on horseback in the morning;' saw when she left the room that the Doctor went down stairs with her; he was gone sufficiently long to take her to the door, as was his habit with patients; I did not perceive anything peculiar in the appearance of this young lady."

The dress-maker, in Third near Lombard-st., referred to in plaintiff's evidence, and the young gentleman, also referred to as having been encountered in the street, here severally testify to the absence of anything remarkable in her manner or appearance. The evidence for the defence then closed, with a long succession of statements by different dentists and their patients, and by physicians, together with a number of citations of printed cases and authorities; all of which went to illustrate the peculiar effects of ether inhalation in producing hallucinations or illusions, and to describe its usual action on the consciousness and will, and powers of locomotion. For the purpose of rebuttal, three witnesses were introduced to prove that ether sometimes prostrates the muscular powers without interfering with the consciousness. An attempt was made, also, to introduce evidence of individual acts of impropriety committed by the defendant, on former occasions, with different persons. This was warmly objected to by defendant's counsel, and not allowed.

The prosecution then closed with the following examination of the family physician of the young lady. This examination is so unintelligible and evidently misreported, as it appears in the Gazette, that we were obliged to consult the witness personally. He was kind enough to put us right, and has enabled us to give it as corrected by his notes.

"Dr. Huston affirmed—I was formerly Professor of Obstetrics in Jefferson College, and am now Professor of Materia Medica and Therapeutics.

Q. What would be the advantage of an examination of a female after her menstrual discharge in disguising or obliterating any marks of violence on the person.

A. The presence of the menses would embarrass an examination very much.

Q. Would it disguise it or not.

A. It would very much mask the appearances. The most positive sign after such an occurrence would be the male secretion about the person of the female; but, unless that were in considerable quantity, it would not be easy to distinguish it, especially if the menses appeared within a few hours, or were very profuse.

Q. Would there necessarily be laceration if a female was violated under the influence of ether?

A. Not necessarily, but it might happen; there would be a relaxation of the parts at that time.

Q. Might there not be connection with a woman without leaving marks of it?

A. Yes. It would depend upon the condition of the parts. Sometimes the muscle that closes the entrance of the vagina is very strong and offers great resistance. I had a lady patient once whose husband called on me for advice, and stated that he had been married six months without being able to have connection. It proved to be a case of the kind, and was relieved by the aid of dilating instruments. Generally, and particularly in females of relaxed habits, no such great resistance occurs.

Q. By Mr. B. Are you Mr. M's family physician?

A. Yes, and have been some twenty years.

Q. Have you examined Miss M. since the alleged assault?

A. No. I was called on by her father, by the request of counsel, as I understood, but, understanding that she complained of no soreness, and that the menses came on very shortly after she left the defendant's house, and had continued ever since, I declined making the examination and advised against it. That was on Wednesday or Thursday of the next week.

Q. and A. Do you know of a letter written by me (Mr. Brown) calling for an examination? I do not.

Q. How long have you been in practice?

A. Nearly forty years.

Q. What effect would ether have in bringing on the menses?

A. It is stimulating, like brandy, and might hasten the appearance of the discharge.

The history of the trial would be very incomplete, without some portions of the able charge with which the case was submitted to the jury. This furnishes the only practical analysis, that appears to have been given; and considering the unsatisfactory nature of the medical evidence elicited, it is as full and fair a survey of the actual ground to be debated, as the prisoner could wish. The few extracts to which our limits must confine us, will exhibit the material points and course of resoning, suggested by the judge as governing the argument on either side.

"One of the learned gentlemen has said that it is an accusation easily to be made, and harder to defend, be the party ever so innocent. For this reason it is usual to look for some corroborating circumstances, which are generally found. If the woman's person discover signs of having had connection, and the act be done in a remote place where persons passing could not be called, all these are, and may be shown in corroboration. Here, however, from the nature of the offence, and the manner in which it was accomplished, none of these corroborating circumstances can be produced. The space of time to which consideration may be directed too is limited; for this offence, if committed, was committed on the morning of the 4th of August, between the hours of 10 and 11 o'clock. The evidence seems to show that she arrived in the neighborhood of 10 o'clock.

No other person was present from that hour to 11 o'clock.

To that point your attention is particularly called, and to what transpired in that room. Give that part of the evidence particular attention and careful scrutiny. The only persons present were the witness, Miss M., and the defendant, Dr. B.

And unless you are convinced of the truth beyond any reasonable doubt you shall at once acquit the defendant. If her testimony, the nature of the circumstances, and her position, cannot be relied on, then there is no other evidence upon which he can be convicted. Her testimony is direct and positive as to what occurred as far as she could judge. And her ability to know what was going on and doing, thus depends upon the situation in which she was at the time. She was under the influence of ether, administered to her in preference to something else calculated to relieve the pain, she being permitted to choose two remedies. The question arises, what effect had it upon her system? Did it deprive the muscular system of power? had she her consciousness? was she able from this consciousness to know what was going on? She tells you that the doctor laid his hands on her, the manner and character of the offence. You are to judge from that testimony, whether the act done was a carnal knowledge of her person. You must be satisfied that the person of Miss M. was actually penetrated by Dr. B.; and you must be convinced of this. The Commonwealth rely exclusively on the accuracy of her statement; they say, and say properly, if she was in a condition to know she could be relied on.

Her character was unimpeachable. And if you find that, at the time of this occurrence, there was no delusion or illusion, then her testimony is to be properly and fairly relied upon.

It is shown to you on the part of the Commonwealth that some of the corroborating circumstances attending a case of this kind, from the very nature of the transaction, are not very satisfactory, owing to the fact that there is relaxation of the body after the inhaling of the ether. An exertion of the muscular power is impossible. The Commonwealth rely on this fact that some of the corroborating circumstances which attend such an act would be wanting, and could not be exhibited from the relaxation of the system. There might not be that evidence which would be exhibited if a resistance had been made."

"On the part of the defence it appears to me the evidence is to be viewed first as to the situation and position of the witness; second, they rely upon the fact that this was an illusion. That at the time of the alleged perpetration she was under the influence of ether, and the fact of its having occurred is an illusion, and entirely unfounded. They perhaps would take another position, and that is, being under the influence of this delusion, and being in a close approximation to her mouth, and the occurrence of this pain at the time, would make some explanation of the point of contact by the defendant. They would probably rely upon this as being a cause of the sensation she referred to, being under the influence of ether, and that the delusion was caused by a pain entirely different from that which took place, and brought on by the position of the woman, being near her monthly changes, they were facilitated by the relaxation of the body. They would, perhaps, rely on the want of corroboration; that no examination of the person or clothes, to ascertain the fact whether the act of connection had been performed. Of course, as Dr. Huston said, if the evidence of the act of connection had remained upon her clothes it would be a strong and overwhelming proof. Although it might to some extent be difficult to determine this, yet science is sufficiently advanced to determine this point. They would probably rely upon the conduct of the lady after this alleged occurrence. This lady came into town that morning, having several ends in view. She arrived at 10 o'clock, after that, there was a visit to the dressmaker, and after that to Mr. T——'s. The defence might have said, in going there she was out of her course, and had the outrage been true it would have been disclosed in the first place to her mother. You will consider this in connection with her position at that time. She was so far in the possession of her reason as to judge what had been done, and either the excuse she makes for not revealing her case to Mrs. P. was entirely untrue, or she desired first to see her mother for that advice so important at that time. They might also take another ground of defence. The improbability that such an offence was committed at that time and place, and upon this they would rely with great certainty; but how, it is for you to say. It is alleged to be committed in the vicinity of a room where there were five persons, who could have heard a scream if made—who could have seen into this room if their attention had been called to it. And therefore the defence say that it is improbable that he would take that time for the perpetration of a deed like this. * * * * *

The last defence they might rely upon would be that of character. They assert it would be highly improbable that a man who has the character of this man would be guilty of such an act. Character, in cases of doubt, is of importance when the evidence leaves a doubt on the mind. When a man even has a character beyond suspicion, it must not be too much relied on."

In reviewing the evidence now before the reader, we must examine it under the two distinct heads of direct testimony and corroborative testimony. Corroborative testimony can not be positive in such a case; and although indispensable to a just conviction, it is of secondary weight, and only valuable for proof according as it approaches the positive in character. If the direct testimony be tainted with a serious doubt, then the corroborative testimony must be doubly strong, in order to counteract that serious doubt. The only direct and positive testimony on this occasion, was that of the young girl herself. The question therefore, to be determined, resolves itself into two inquiries: 1. Is the evidence of the complainant to be relied on beyond a reasonable doubt? 2. Are the circumstances sworn too in corroboration of the complaint sufficiently decisive to confirm her evidence beyond a reasonable doubt?

Now let us analyse the plaintiff's story. The time to which it especially refers may be divided into three separate and successive periods. First, before the etherization; second, during the etherization; third, after the etherization. Her veracity and mental sanity are not suspected. There is no reason then to disbelieve her account of period No. 1. It is clear enough as to the general nature and succession of events, although not remarkably precise; but as to lapse of time and its special distribution, the narrative is not so very clear. She reaches the house about ten o'clock, after having walked, she can't tell how far, with her betrothed from the railway station, which she had left at half past nine. She is detained "a few minutes" and then goes up stairs into the office. Here "a few minutes" of time pass in common-place conversation, while she takes off her bonnet and seats herself, and he washes his hands. "In a few minutes" the operator tells her he must speak to one of his men, gives her a magazine and leaves the room. She does not know how long he was away, nor is this shown by other evidence. It is only evident that neither operator nor patient was disposed to be in haste. There was not enough to do. He returns, finds her in the chair, goes to the instrument case, and at last commences on her tooth. She does not say how long he pottered at the instrument case or how long he was scraping at her tooth. Here we need important information. What is his habit? Is he slow and desultory, or methodical, prompt and rapid? How long does it usually take him to clean and fill a very carious tooth? Where is the tooth itself that was the unwitting source of all this trouble? He seems to have loitered most unfortunately during a considerable portion of that eventful or most uneventful hour. Who can say that he did not loiter still more fatally until the hour expired? If there were time and to spare for any operation on the teeth, surely there was more than time enough for the perpetration of the single momentary act, so vaguely charged against him. Was it his habit to adhere so rigidly to a single specified procedure on a set of teeth that had been under his professional care throughout their possessor's life? Had he no right, if in the course of operating, it were deemed advisable, to substitute extraction of a tooth for plugging or to remove some other tooth that might be ascertained to be too far gone to be preserved without injury to its neighbors? We regret that none of his professional brethren were called upon to throw, perchance, some light upon these problems in dentistry, and that some of his numerous friends and vouchers were not interrogated in regard to his usual *modus operandi*. To return to Miss M. The operation, whatever it might be, was painful? this leads to a consultation, a determination to resort to ether; another long or short delay, and the administration of the vapor from a napkin. Sooner or later she feels dizzy, cold and numb; her eyes are closed; she continues breathing the ether and the symptoms "grow upon her." The mysterious spell is deeply working, yet she does not lose her "consciousness of what is doing;" she is aware that he is taking liberties which would rouse any woman in her senses, yet she makes no effort to unclose her eyes, and is incapable of outcry or resistance! Here we must pause, already past the threshhold of the second and crowning scene of this wretched drama. At first dizzy, cold and numb, she has become powerless, voiceless, sightless and yet feels the slightest touch, perceives a breath, hears every footfall and even suffers pain, one throe of which receives the worst interpretation. This is one case of a thousand—a miracle of sense and nonsense, even for that juice of Oberon's flower, the wondrous ether!

On her own showing, afterwards confirmed by other witnesses, she had been inhaling ether, and she accurately describes the ordinary gradual invasion of the ethereal aura.

Now if we are to believe the first part of her recollections when she was undoubtedly awake, we are bound, according to the well established experience of the effects of ether, to believe that she had then become confused and bewildered, and liable to hallucinations and disordered sensations, if not altogether unconscious—*non compos* in other words—and for the time being as incapable and incompetent a witness as a lunatic, an inebriate or an idiot. We know that there are grades of competency even for these afflicted classes, and that she may have passed through different grades of consciousness herself, but how are we to determine this? Her own senses were fallacious, and there is nothing else to aid us in the details of her story. Nor can we find anything in the evidence, or on record, or in our own experience, that will justify us in the admission, that she or any one had ever been *so helpless* under the influence of ether, and yet *entirely* aware of what was going on.

A similar assertion as to consciousness may be heard in almost any drowsy company, and with quite as much reason, for aught proved to the contrary, although the self-styled "wide awakes" may have been snoring all the evening. Nothing is more common than this illusory insomnia among invalids at night; and it is well known to amount often to a most absurd hallucination. We have had patients gravely to assure us in the presence of a crowd of students, that they "had not slept a wink for six weeks!" others that they never closed their eyes at all; and we have heard of an individual, formerly well known in Philadelphia, who used to tell every body that he had not slept for years. It is in this half asleep and half awake condition that dreams in the sane, and hallucinations or illusions in the insane, are most frequently and vividly perceived. The topic is an interesting one, but we cannot dwell upon it further. The admirable monographs of Michéa, Baillarger, Briére de Boismont and Calmeil, probably contain the best appreciation and expose of such strange intellectual vagaries. We greatly doubt the genuineness of these exceptional cases of perfect or even tolerably clear intellectual consciousness with abolition of the will and muscular power. They have not yet been fairly tested. The difficulty of establishing the fact of lunacy in a great variety of cases, where weeks and months of observation are allowed, is a lamentably common source of error and confusion. How then is it possible to detect a want of mental equilibrium in the fleeting visions of an anæsthetic aberration that comés and goes like a delirium or a dream? But it is still harder to comprehend the possibility not only of perfect consciousness, but of the sense of pain and touch in association with loss of muscular force and will. That she could actually suffer pain or become aware of outrage while deprived of the ability to move a limb or make the least outcry or resistance, is by no means in accordance with the well ascertained order of sequence in the stages of anæsthesia in the vast majority of instances. If anomalous cases of that kind have been authenticated they are too rare to make a rule, or to free our own from an unwieldy load of doubt. We might cite a multitude of authorities in support of this position. Channing, Flagg and others of this country; Snow, Simpson, Robertson, Forbes, Ranking and others of Great

Britain; Longet, Flourens, Velpeau, Malgaigne, Dubois, Sedillot, Bouisson, Bayard, as well as many others, French, German and Italian, whose works are noticed in the different journals, might be quoted. We are glad, however, to be spared the necessity of dwelling on these and other points relating to this branch of the discussion, by the able paper of our fellow collaborator in the present number of the Examiner. The reader will there find the medico-legal bearings of the psychological phenomena of etherization fully and forcibly depicted. We take great pleasure in recommending the note of Dr. Stillé to general consideration.

If the plaintiff happened to be so keenly alive to what was going on around her, she must certainly have been aware of more than she describes. It is strange that she was not more closely questioned. A false delicacy in her case, on the part of the cross-examination, was surely a mistaken kindness, if it were allowed to operate; since an opposite course might have resulted, as we are inclined to think it would, in the strongest evidence of still unsullied innocence, unless some indiscreet domestic inquisition had succeeded in enlightening her. Much has been said about "that hour." Can she really remember nothing of the second period of time, be it long or short, but what is contained in her rudimentary sketch? The outlines are deep and broad, perhaps, but they seem dimly and dismally far apart! We mean no indelicate allusions. We feel the most earnest desire to spare the feelings of all parties, and of none more than the unfortunate subject of these comments. We wish to believe her free from soil, and if we strike rudely at the ill stained slough that has been thrown around her purity of fame, it is but to destroy its every trace.

But we refer to all that happened, and to much that no woman would hesitate to speak of. How does she *know* what he was doing at the time? Admitting for the moment that the pain was not imaginary, and that she did feel his breath, might not that pain have suggested the dream and a struggle at the waking moment? We mean not an "erotic dream." No such dream or sensation is admitted by the plaintiff, although such dream and sensation have certainly occurred in many cases. We have heard several cases individually related, and we could quote others as well as those presented by Dr. Stillé. We might narrate a striking case of erotic hallucination occurring without ether in an undoubted virgin, narrated in detail by M. Michéa, (*Hallucinations, Mém. de l'Ac. Royale de Méd.*, vol. 12, 347,) but we have neither space nor inclination for such displays. We might challenge, too, the experience of every one accustomed to the treatment of the insane, and could easily draw upon our own recollections for many disagreeable reminiscences which would be very pertinent in this connection, but we cheerfully forbear.

Agreeing then for the moment to the occurrence of the pain, and attributing it to innocent and natural causes, we do not admit that at the time the plaintiff had any idea, certainly any clear idea, that she was being ravished. That was probably an after thought, and for aught that appears to contradict this view, the long drawn conclusion of a whole day's brooding. But more of this in discussing the history and "phenomena" of period No. 3, the last sad scene of our semi-tragedy.

He did not, as she remembers, "operate on my tooth after he had given me the ether." Now who is to determine this? The only witness present was, according to her own admission, under the influence of ether. Her eyes were closed; how does she know that he went round before her and raised her clothes? Was she really clairvoyant? She could not see what was his position; she did not feel his person against her person, and yet because she felt pain in a certain part, she was sure that he was penetrating that part! She felt his breath, but no part of his limbs or trunk; how then does she know that he was not behind her, supposing, for the moment, that she did not err in the sensation? How could she in her utter ignorance and inexperience distinguish the pain of penetration from any other kind of pain in the sexual organs?

How can she be sure, that at the very moment when some internal action may have so developed a predominant pain in the vagina or its annexes, he was not still looking in her mouth and working at her defective tooth? How does she know that she did not herself unfold her limbs, slip down upon the chair, and herself unconsciously push up or throw up her clothes at the very moment when some hysterical or other spasm, under the combined influence of subsiding etherization and approaching menstrual paroxysm, was producing the single pang which she interpreted so seriously? How can she tell that this fatal pain was not the mere effect of the monthly change which she was expecting at that very hour? Did she never feel such pains before? What was her usual habit? We might dwell upon this point, but time presses, and we are writing for readers who need no aid in such inquiries. Would it be likely that Dr. B. should leave her, go to the wash stand and wash his hands without previously replacing her and her clothes in decent order if he had been the guilty violator she supposed? If he were working at her teeth when such a movement had occurred, what more natural than for him at once to cleanse his hands for the purpose of righting her disorder, as she describes him to have done when she was coming to her senses and stole that reconnoitring glance?

Women of undoubted virtue have been known to pull up their clothes and throw themselves on sofas while under the influence of ether, and they have been known to make even stronger demonstrations, as well as others less equivocal. We do not mean to insinuate that in this case any thing of that kind occurred. It is not necessary. We have no objection to admit the fact of pain, and to allow that it may not have been produced by sexual excitement; but all medical men will understand, that such pain could be more easily and naturally accounted for, without this intervention of the ill defined and far-fetched phantom conjured up by a deranged imagination, and fostered by a kind but wofully mistaken sympathy.

Supposing the act attributed to the defendant were fairly attempted or completed, or even partially completed, it is impossible to assume that the alleged victim knew exactly where he was and recognized his breath when she perceived the pain, without taking it for granted, that she at the same time felt other parts of his body and limbs more or less in contact with her body and limbs, and that she noticed other acts of his concomitant with that of penetration. Yet she expressly denies the sense of contact, and is not reported to have said a word in explanation beyond the extremely meagre statement of a puff of breath at one end of her body, and a twinge of pain at the other end, which have by a stretch of judgment, any thing but charitable, been twisted into the definition of a very different process. To demand a detailed and graphic picture would be cruel and absurd. No decent, virtuous woman could afford such reminiscence even where the sense of wrong were not involved. It is not a time for a self possession that would deliberately note the current of events. We admit that women are mistaken with faculties unclouded by ether or any other drug. We admit that they may think themselves deflowered although they really have escaped; and, *vice versa*, may consider themselves safe when they have actually been debauched. Still she claims to have a "distinct memory" for impressions which she names without describing. We take no account of the feeling of the pulse, the touching of the arm, of the bosom and other parts of the person. These acts do not fill up the time required to be disposed of. The cleansing of the carious tooth would have occupied more time; and then the "liberties" are such as other patients might be proved to have imagined under similar circumstances. She proves too little or entirely too much. In order to effect a conviction on such evidence, the vital part of the question must be begged. The jury are called upon to accept the proof of etherization—as every body does; to ignore the probability of the most frequent if not customary, effects of ether on the plaintiff—which every body will not do; to regard the action of the ether on the plaintiff as exceptional, and, therefore, not invalidating the directness of her testimony—which many would not dare to do; and lastly, to fill up the void in the only material evidence produced, by dint of their imaginations—which they ought to be ashamed to do! Verily, a lame and impotent conclusion, if there be no adequate corroborative proof at hand, to cast the shadow of a reason on this confusion worse confounded!

What, then, does the corroboration really amount to? This question brings us to the study of the third period of time in the stages of our investigation; to the sifting of what happened or did not happen, after etherization had properly subsided.

Here occurs a seeming irregularity in our chronological arrangement. "That tooth" is not yet "sacrificed." She is once more invited to take the subtle fluid and, nothing loth, agrees. There is no assault this time, no loss of power—in short, no anæsthesia, for she feels the wrenching of the tooth and screams so loud that she is heard down stairs. She has again become a competent witness. Other witnesses look in from time to time according to her account, confirmed by theirs, and finally another patient is brought up. Do we hear from either of these new actors in the scene, or from the sufferer herself, of any distress of mind or body, such as would be certain to overwhelm most women under such a fearful trial? Not one word! She is calm and complaisant, converses freely, and suggests and makes a future appointment with her supposed destroyer! What matron or maiden in the land will say that such self command and entire absence of emotion are natural, whether or not under the necessities of doubt and fear? We can only account for it in any degree by the hypothesis of a prolonged partial intoxication by the ether, such as frequently occurs. Still in the clearness of her recollection, as sustained by the evidence of Mrs. P., she betrays no confusion of mind or internal conflict of feeling; she sets out upon the business of the day as she would go from the breakfast table. In attending to her errands, she takes a long walk through the most frequented streets, stops to regale herself with ice cream at one of the most popular confectionaries, then fulfils an engagement to dinner at a friend's. She accompanies her host and hostess on a drive in the afternoon; and although eating little and looking sick at the dinner table, it is not until they sit down to tea that her doubts reveal at once their vent and climax, and the dreadful myth assumes a form and being in the private consultation with Mrs. T. Dr. B. appears to have thought that the ether was "still in her" when he advised her to walk rather than ride a mile and a half or two miles to the dress maker's and back to Mrs. T's. Would he have given this advice, and could she have followed it without great difficulty and pain, if the mischief had been done to her that is laid to his account? Many girls in such a stress would have required an ice cream saloon at every crossing. We do not deny that this is not the invariable consequence. It is the usual one, however, and its absence in this instance only increases the deficiency of corroborative proof.

It will be useful here to institute a comparison between the affair of Dr. B. and that of the Parisian dentist, the only other known to us. This individual was convicted Oct. 30, 1847, of having violated two young girls on two successive days. A brief account of one of the cases, that of Henrietta Soyard, found its way into the journals at the time, and is given below; the other is noticed only in the partial report of the *résumé* of the presiding judge, portions of which we have extracted from the *Abeille Médicale.*

"Lundi, une jeune personne attachèe á un magasin du quartier du Palais Royal 's'était rendue chez un dentiste pour faire extraire une dent. Le dentiste l'engagea á la faire plomber seulement; et comme sa cliente redoutait la douleur, il lui proposa l'emploi de l'ether, qui fut accepté. Que se passa t-il pendant que cette jeune personne était sans connaissaince? Il serait difficile de le dire; toujours est-il certain qu'en sortant de chez le dentiste au bout de trois heures, la jeune fille était dans un *dèsordre affreux.* La dame de son magasin ne put se rendre compte de cette longue absence et de l'état dans lequel se trouvée cette jeune personne. Celle-ci malgré l'anéantissement causé par l'ether, conservait quelque sentiment des outrages qu'on lui avait fait subir, et quelques mots qu'elle laissa échapper font naitre des soupçons; on la fit mettre au lit, et un médecin ayant été appelé, constata d'une manière évidente les violences qui avaient été exercées sur sa personne. Une plainte a été déposée, et l'auteur de cet acte odieux a été arrêté et remis á la disposition du procureur du roi."—*Gaz. Méd., Juillet,* 1847.

This young girl retained a troubled recollection of the outrage which her subsequent agitation and the appearances revealed at the medical examination amply corroborated. The following extract from the judge's charge on the trial, still further confirms the justice of the accusation on both indictments. "Remarquons ensuite les détails accessoires. Quand ces jeunes filles se présentent, comment l'accusé entame-t-il la conversation? Par des propos indiscrets; 'Avez vous des amans?' dit il á l'une des jeunes filles. 'Etes vous vierge? Avez vous été mère?' Pourquoi ces questions, quelle pouvait en etre l'intention? Ne voit on pas le germe de mauvaises pensées, d'infames désirs. La jeune Bazin vous a rendu compte des actes dont le souvenir lui était resté et qui lui ont donné la conviction de la violence et de l'outrage dont elle a été victime: elle sentit ses forces paralysées, ses membres s'engoudir et se trouve dans l'impossibilité d'opposer une résistance active, energique a l'action de l'homme qui se trouvait devant elle; mais quoique ses forces fussent épuisées, elle ne perdit pas cependant completement l'intelligence et la perception des actes qui furent accomplis sur sa personne, et son trouble ne fut pas au point qu'elle n'en pût conserver la mémoire. M. le President analyse ici la dèsposition tres significative de la jeune fille; puis il insiste sur l'étrange coincidence des faits arrivés aux deux plaignantes à un jour d'intervalle. Examinant ensuite la question de savoir si les déclarations de ces jeunes filles ne pourraient pas être le résulte d'une hallucination, d'un rêve produit par l'éther, M. le presidente enumère les circonstances de fait que le ministère public a fait valoir pour combattre cette hypothese; il rapelle les cris jetés par la jeune Bazin, ses larmes, son desespoir, les injures qu'elle addressait au sieur Aimé, qu'elle traitait d'infâme; puis, d'un autre côté, les réponses embarrassés de l'accusé."—*Ab. Méd.*, 332, 1847.

The experience of these young persons approaches in resemblance that of Miss M. *only in their ineffectual resistance.* In attendant circumstances theirs differ very much from hers in being far more true to ordinary nature.

It is unfortunate that Mrs. T., the witness first examined in regard to the conversation at the tea table, was not allowed to give her own account of the private interview in which the glimmering suspicions were first brought to light and perchance reduced to ultimate development. Leading questions are so apt, in spite of best intentions, to form the staple of such revelations; and subsequent impressions and suggestions are hence so liable to be mixed up with genuine facts, that it behoves us, as medical advisers, to receive, with extreme deliberation, all evidence of this kind that has passed through the crucible of home. Mothers and guardian matrons may easily take alarm, and by unfounded and hastily indulged complaint involve themselves and their dependent children in endless grief.

On this topic we might refer to numerous authorities, and, if there were room for it, would be glad, in illustration of our position and its grave importance, to quote from a late admirable paper by Mr. Wilde, of Dublin, in which five cases of "alleged felonious assaults" on children affected with vaginal discharge, recently tried in Dublin are thoroughly discussed. (*Med. News and Libr.*, 1853, Nos. 130, 131, 132, *from Lond. Med. Times and Gaz., Sept.* 10, 1853,) Beck, (*Med. Jurisp.*, 1, 159,) is remarkably full on this point, as he is in everything else of interest.

It does not appear that Mrs. T. either made or proposed any kind of local inspection. The most superficial examination of the injured parts and of the linen would have been invaluable at that early date. A regular professional investigation might have been conclusive. It was not too late, at any time before the trial, to ascertain the fact of a healthy and unimpaired integrity of the vulval region, altogether incompatible with the idea of the painful connection sworn too by the plaintiff. It is hard to conceive of such painful penetration, without the consequent production of a certain amount of physical injury and alteration and temporary soreness, that might have been distinctly recognized within the first three days, if not at a later period.

Much change or permanent structural alteration, we admit, is not invariably to be expected from a single, and more or less imperfect act; but objections of this kind are negative only, and too conjectural to justify omission of a test so generally available, often so positive and always so obviously important to all concerned.

If there be any local or constitutional peculiarity that would be likely to diminish the value of the usual signs, such peculiarity can be better ascertained and estimated through an exploration than without it. The presence of the menstrual flow would, of course, embarrass such an exploration, and render it more unpleasant to patient and physician; but it would not necessarily destroy its value. An examination at any time, in an inquiry that concerns the possible condition of a part, is certainly better than none at all. Nor is there any positive experience to show that the effect of ether is to relax the vagina, fourchette, hymen or any tissue but the muscular, except, perhaps, under the peculiar influence of childbirth. The hypothesis of relaxation of the sexual region in the virgin, or at least in the particular individual concerned, might be easily tested by making the desired inspection, while she were subjected to the influence of the supposed relaxing medium; she would thus be spared much of the distress inseparable from the exposure, while the conditions would certainly afford the best criteria for ascertaining the requisite truth. In a word, if the parts had become dilated and relaxed under the menstrual flux, an examination would have proved this to a demonstration. So, also, with the ether; if that agent had produced a relaxation at the menstrual period, its administration would have had the same effect again. We have not hitherto suggested the possibility of partial penetration only, of vulval violation, because such half-way action could not have brought about the pain, upon which the whole accusation hinges. For the same reason we attach little weight to the idea of relaxation. Either supposition but disposes of one horn of the dilemma, and ought rather to favor the defendant than the plaintiff. The third cardinal rule of Beck, in these investigations, avers that "a speedy examination of the parts is all important in disputed cases." (*Op. Cit.* 1, 163.) He directs, too, that the body of the male also should be inspected, and that the male organ should be examined for obvious purposes of comparison. These are the rules of all authoritative medical jurists of the present day. They should be insisted on as absolute under all circumstances and contingencies; and the neglect of their observance ought always to be regarded as a radical injury to the cause of true justice.

Not less indispensable to the right understanding of doubtful cases, is the corroboration to be derived from an examination of the stains, upon the back and front portions of the chemise, of blood, of serous oozing, and of spermatic fluid. We do not pretend that the inquiry would be easy or conclusive in the present case, but it might have been so, and no one can prove the contrary. The sole evidence on this point is the mother's, and she swears only to an unusual amount of the customary monthly flow, just what the ether might produce. Instead of being hurried to the wash tub, that linen should have been put under formal seal, and carefully subjected to the usual chemical and microscopical investigations. (See *Devergie, Méd. Lég.* 1, 143.)

Where the direct testimony is so exceedingly imperfect in its form, and where the only material part of it is so strongly suspected of unsoundness, every jot and tittle of corroborative evidence should have been religiously accumulated and preserved. Yet there is none to show—nothing but a poor apology for emptiness. There was a lion in the way of an examination—the old romance about the rudimentary hymen; the bugbear of its destruction by disease, by accidental rupture, by foreign bodies; its presence in some pregnant women, its absence in some virgins.

Conditions of barely possible occurrence, anomalous if not apocryphal, are dragged forth to mystify deductions based on the popular experience of ages, and upon the soundest medical authority. "*Il ne faut pas voir en Médecine légale, la virginité morale mais bien la virginité matérielle. . . . 1, Si la membrane hymen existe, la défloration n'a pas eu lieu; 2, si elle n'existe pas, la défloration a dans les neuf cent quatre-vingt-dix-neuf centièmes des cas. été opérée. (Devergie, Méd. Lég.* 1. 135.) If the hymen is not in its place, defloration has been effected in nine hundred and ninety-nine cases of a thousand. So says one of the highest authorities in medical jurisprudence.

These speculative difficulties, were they listened to, would paralyze all progress. They only increase the necessity for stricter observation. They might embarrass the defence, but could not strengthen the prosecution. Further special reference on this and kindred topics might interest some readers, but the patience of many others, like our own, and the already liberal space allowed us in these pages, must be by this time so nearly gone, that we may content ourselves with pointing to the books before us. We have now on our table the admirable works of Beck, Guy, Taylor, Devergie, Briand and Bayard, as well as some others of inferior note; and we have consulted still others which are not in our possession, but no useful purpose can be served by a parade of erudition while ample authorities are, or ought to be, within the reach of every medical reader. With Beck, Taylor and Guy at his hand, the careful practitoner of this country may easily prepare himself to meet most ordinary cases, and may be enabled to clear up many painful misapprehensions, and save more than one family from hopeless anguish and disgrace. It is not long since we were consulted by the mother in a case of suspected connection with a child of ten years, in which an innocent man had been implicated, and would have been dangerously involved, but for the accident that brought the child before a surgeon. If the attention of our readers should be properly awakened to the study of these questions, and to the fact of their continued frequency, and scarcely less frequent want of just foundation, the object of this paper will be gained. It was the remark of Prof. Amos, years ago, says Taylor, that for one real rape tried in the circuits there were on the average twelve pretended cases! The good old rule in murder is fearfully reversed in rape; ten unoffending men may suffer lest one guilty may escape. Who has not heard how easily the charge is made, and how hard to be repelled? As it was in the time of Hale, so has it ever been; what was a truism in those hanging days is a proverb now. Such calumny sears all alike; there is none too pure or strong to come within its grasp; it palsies defence; yet few can realize the terror of this truth. Let the reader imagine himself before a court and jury. His professional advisers may whisper that science will protect him, but he knows how often it has proved a broken reed. Friends, Christian and worldly, may gather round to cheer him, but their hope-inspiring accents are lost in the storm of sneers and slang which assails even the habiliments of the wise and good in such a cause. Wherever he may cast his weary eyes he sees the slow and moving finger too surely pointing out his doom. A few disconnected words, a single positive assertion, however flimsily sustained, however ignorant the witness, may work more certainly upon that motley company than the clearest reasonings of science or the best established abstract truth. Should this fancy overtask our reader's nerves, let him follow the unfortunate to his penitentiary cell, and watch him, as we did years ago, slowly dragging to the grave a wreck of mind and body. We have seen a tottering old man of over sixty, infirm beyond his years, serving out a ten years' sentence on an indictment, which, in his case, was a manifest and foul absurdity. We could recall other less striking instances, and we doubt not, from our experience as officer and visitor of prisons, that few penal establishments of any size are unprovided with their sickening array of similar victims to the commonwealth's prerogative.

But we are told of insufferable danger threatening alike our daughters and sisters, wives and mothers; a "terrible example" is demanded, and we are asked to immolate a family as a holocaust to their imprudence and our fears! The answer is an easy one; let them not go into the lion's den alone. Let the hydra-headed beast be muzzled, let it be a criminal offence to administer chloroform or ether to any one, except in the presence of a relative or friend of similar sex. Let public opinion, more potent than the laws, enforce this rule on all occasions, and the demon of opportunity is gone forever.

